# Genome wide analysis of sex difference in gene expression profiles of bone formations

**DOI:** 10.1186/1471-2105-15-S10-P3

**Published:** 2014-09-29

**Authors:** Yue Huang, Xiaodong Zhu, Lishi Wang, Xiaoyun Liu, Robert W Williams, Lu Lu, Yan Jiao, Weikuan Gu

**Affiliations:** 1 Department of Orthopedic Surgery and BME, University of Tennessee Health Science Center, Memphis, TN 38163, USA; 2 Mudanjiang Medical College, Mudanjiang, 157001, China; 3 Department of Anatomy and Neurobiology, University of Tennessee Health Science Center, Memphis, TN 38163, USA

## Background

Geber and Murphy [1] found that a sex and stature bias is evident among adults in which males and taller individuals displayed statistically significantly higher levels of scorbutic lesions. Thus the study provided evidence to support an investigation on the gender difference with a lack of vitamin C (VC) in humans. In an animal study, a sex difference has also been evidenced [2,3]. Previously, we have studied a spontaneous bone fracture (*sfx*) mouse [4] which lacks the gene for L- gulonolactone oxidase (*Gulo*), a key enzyme in the ascorbic acid (AA) synthesis pathway. In this study, we investigated the gene expression profiles between female and male mice using the *sfx* mouse model and BXD RI strains. The objective of this study is to identify sex differentially expressed genes in bone using the *sfx* model and BXD RI strains.

## Materials and methods

We first identified the genes that are differentially expressed in the femur between female and male *sfx* mice. We then analyzed the potential gene network among those differentially expressed genes with whole genome expression profiles generated using spleens of female and male mice of a total of 67 BXD (C57BL/6J X DBA/2J) recombinant inbred (RI) and other strains.

## Results

By analyzing the female and male mice separately, we found many more differentially expressed genes between wild type and *sfx* mice from either female or from male mice than we found previously using RNA of a mixture of female and male mice. It is obvious that the skeletal system is different between female and male. The female and male skeletal system is most likely to react to the VC deficiency in *sfx* mice differently. The comparison between disease and control samples using data of sex mixture suffers from neutralization of gene expression levels between the female and the male. This result suggests that in the study of genes that are potentially affected by the sex or the gender, data from female and male individuals should be analyzed separately. Many reports have been using the sex balanced data of mouse strains.

## Conclusions

Our data posed a question about whether the balanced data should be used without understanding of the sex differences.
